# Multiorgan eosinophilic infiltration after initiation of clozapine therapy: a case report

**DOI:** 10.1186/s13104-017-2662-1

**Published:** 2017-07-25

**Authors:** Dorota Marchel, Anita L. Hart, Patricia Keefer, Jennifer Vredeveld

**Affiliations:** 1 Pediatric Primary Care Clinic, Rutledge Tower, 3rd Floor, 135 Rutledge Avenue, MSC 560, Charleston, SC 29425 USA; 2 University of Michigan Health System, 1500 East Medical Center Drive, Room 3119 TC, Ann Arbor, MI 48109-5376 USA; 3 University of Michigan Health System, 1540 East Medical Center Drive, SPC 4280, Mott 12-525, Ann Arbor, MI 48109-4280 USA

**Keywords:** Case report, Clozapine, Eosinophilia, Eosinophilic infiltration, Multiorgan dysfunction

## Abstract

**Background:**

The eosinophilic response to clozapine is well described in the literature, causing a variety of responses, from serositis to colitis. However, there are not case reports describing a clozapine-induced marked eosinophilia resulting in multiorgan dysfunction.

**Case presentation:**

In this case report, we describe a 24 year old Caucasian male who presented with severe systemic eosinophilia resulting in eosinophilic GI tract infiltration, myocarditis, pericardial and pleural effusions with dramatic improvement following drug withdrawal.

**Conclusions:**

Clozapine associated eosinophilia should be suspected in the setting of eosinophilic infiltration of multiple organs.

## Background

Clozapine is an atypical antipsychotic medication known to have multiple hematologic side effects, most seriously agranulocytosis, but also notably eosinophilia. Clozapine-associated eosinophilia has been reported in two different forms: transient benign eosinophilia and eosinophilia with end organ damage [1–3]. Clozapine-associated eosinophilia with end organ damage has reportedly been associated with pancreatitis [4], pleural effusions [5], eosinophilic pneumonia [6], colitis [7], hepatitis [8], and pericarditis [9]. Here we describe a patient with a dramatic eosinophilic response to clozapine resulting in systemic manifestations.

## Case presentation

A 24 year old Caucasian male with a history of asthma, atopic dermatitis, and schizoaffective disorder was started on clozapine therapy for persistent auditory hallucination after trials of numerous other therapies including olanzapine, risperidone, aripiprazole, ziprasidone, haloperidol, and fluphenazine. Clozapine was added to an existing regimen of paliperidone, benztropine, divalproex sodium, and escitalopram. About three weeks after initiating therapy, he was admitted to a hospital with nausea, vomiting, cough, chills, and chest pain. Evaluation during his admission revealed the following: computed tomograph (CT) of the chest showing diffuse bilateral hazy opacities with small bilateral pleural effusions; echocardiogram with tachycardia, mildly decreased left ventricular systolic function with an ejection fraction of 45–50%, moderate to severe hypokinesis of the right ventricle, and moderate pericardial effusion without evidence of tamponade. Laboratory evaluation showed normal electrolytes, renal function, and hepatic function. White blood cell count was 14.7 K/μl with an absolute eosinophil count of 1.5 K/μl. It was thought that symptoms most likely represented pneumonia with viral pericarditis and viral myocarditis. Medication additions at discharge included levofloxacin for pneumonia, colchicine for pericarditis, and lisinopril and metoprolol for myocarditis. His psychiatric medication regimen was not changed.

The day following discharge he presented with profuse, watery diarrhea complicated by dehydration and acute kidney injury. Abdominal exam on admission was benign. White blood cell count on admission was 21.2 K/μl with an absolute eosinophil count of 2.9 K/μl. Liver function tests were normal, no rash was present, and he remained afebrile. Colchicine was stopped, but diarrhea continued. The patient revealed that his source of water is a fresh water stream. However, evaluation for Giardia and parasites was negative, as was evaluation for clostridium difficile, human immunodeficiency virus (HIV), cytomegalovirus (CMV), cryptosporidium, and Churg-Strauss. The inpatient psychiatric team was consulted shortly after admission to assess the need to stop clozapine in the setting of eosinophilia. They were initially reluctant to stop clozapine given the patient’s significant psychiatric improvement on clozapine, prior failure on multiple other medication regimens, and possibility of parasitic infection while work-up was pending. During his hospital stay, his eosinophils continued to increase with a peak absolute eosinophil count of 19.1 K/μl. Additionally, esophagogastroduodenoscopy and colonoscopy biopsies revealed diffuse eosinophilic infiltration of the esophagus, gastric antrum, duodenum, ileum, and colon. With the guidance of the psychiatry consultants, clozapine was subsequently tapered off while paliperidone was increased. Benztropine, divalproex sodium, and escitalopram were continued. He tolerated this medication adjustment well without return of his hallucinations. As his eosinophilia improved, his diarrhea resolved. On 1 month hospital follow up, eosinophils had decreased to an absolute count of 1.1 K/μl.

## Conclusions

Adverse hematologic side effects of clozapine can be severe, with agranulocytosis being most well-known. However, clozapine associated eosinophilia can also be significant. The incidence of eosinophilia associated with clozapine has been reported from 0.2 to 62% [1, 3]. While there are many reports of transient, benign eosinophilia following initiation of clozapine [1–3, 10], eosinophilia can also be severe and result in end organ damage. The degree of eosinophilia in this patient is suggestive of a marked immunologic response and resulted in diffuse eosinophilic intestinal infiltration. Additionally, his initial presentation with pleural effusions, myocarditis and pericarditis was likely due to drug induced eosinophilia, rather than a viral etiology as previously thought. With eosinophilia involving multiple systems, drug reaction with eosinophilia and systemic symptoms (DRESS) syndrome could be considered. However, this patient did not have rash, lymphocytosis, thrombocytopenia, elevated liver function tests, lymphadenopathy or fevers as would be expected with DRESS [11].

As patients on clozapine typically have difficult to control psychiatric symptoms and have often failed or poorly tolerated several other agents, there may be some initial reluctance to stop clozapine due to eosinophilia, especially as this is often benign and transient. However, if there are signs of end organ damage, clozapine should be discontinued with the guidance of a psychiatrist. This case provides a rare example of severe clozapine-associated eosinophilia affecting multiple organ systems with histopathologic evidence of this effect and response to withdrawal of therapy.

## Abbreviations

CT: computed tomograph
HIV: human immunodeficiency virus
CMV: cytomegalovirus
DRESS: drug reaction with eosinophilia and systemic symptoms

## References

[1] Aneja J (2015). Eosinophilia induced by clozapine: a report of two cases and review of the literature. J Family Med Prim Care..

[2] Tihonen J, Paanila J (1992). Eosinophilia associated with clozapine. Lancet.

[3] Wysokinski A, Kolinska J (2015). Rapidly developing and self-limiting eosinophilia associated with clozapine. Psychiatry Clin Neurosci.

[4] Garlipp P (2002). The development of a clinical syndrome of asymptomatic pancreatitis and eosinophilia after treatment with clozapine in schizophrenia: implications for clinical care, recognition and management. J Psychopharmacol.

[5] Patel J (2012). Clozapine-induced peripheral and pleural fluid eosinophilia. Ann Pharmacother.

[6] Hashimoto N (2015). Simple pulmonary eosinophilia associated with clozapine treatment. J Clin Psychopharmacol.

[7] Karmacharya R, Mino M, Pirl W (2005). Clozapine-induced eosinophilic colitis. Am J Psychiatry.

[8] Thompson J (1998). Hepatitis, hyperglycemia, pleural effusion, eosinophilia, hematuria, and proteinuria occurring early in clozapine treatment. Int Clin Psychopharmacol..

[9] Kortner K (2007). Eosinophilia indicating subclinical clozapine-induced pericarditis. J Clin Psychiatry.

[10] Majumder P (2011). Clozapine induced eosinophilia. Indian J Psychiatry..

[11] Choudhary S (2013). Drug reaction with eosinophilia and systemic symptoms (DRESS) syndrome. J Clin Aesthet Dermatol..

